# Volatile light hydrocarbons as thermal and alteration indicators in oil and gas fields

**DOI:** 10.1038/s41598-024-63100-0

**Published:** 2024-06-03

**Authors:** Khaled R. Arouri

**Affiliations:** Saudi Aramco, 31311 Dhahran, Saudi Arabia

**Keywords:** Crude oil, Energy science and technology

## Abstract

Volatile light hydrocarbons (VLH) are an essential component of reservoir petroleum fluids. Understanding of their origin and fate is crucial not only in exploration but increasingly also in petroleum engineering, as this greatly impacts fluid typing, proper mapping, recoverability and economic value. Due to their sensitivity to subsurface thermal stress and geological alteration processes, their proper characterisation holds promise to understanding the thermal conditions under which petroleum fluids were generated and subsequent fluid modifications during migration and within the reservoir. To study the behaviour of these hydrocarbons under different geological conditions we selected oil and gas fields from two giant conventional petroleum systems in the Arabian Peninsula collectively spanning the entire petroleum spectrum from heavy oil to dry and sour gas. In situ representative bottomhole or recombined pressure–volume–temperature (PVT) fluid composition data were constrained with molecular and stable carbon isotope geochemistry in key wells. Systematic covariance among the slope factor (SF) of propane to pentane and the isomer ratios of butane and pentane with reservoir engineering and geochemical variables in well-constrained black oil to gas condensate petroleum systems allowed the derivation of three formulas to calculate thermal maturity in terms of vitrinite reflectance equivalent from VLH fluid composition: (1) %VRe(SF) = 0.38 SF + 0.41, (2) %VRe(i4) = 1.70 (iC_4_/nC_4_) + 0.61, and (3) %VRe(i5) = 0.89 (iC_5_/nC_5_) + 0.56. The slope factor, iC_4_/nC_4_, and iC_5_/nC_5_ ratios all increase monotonically with the thermal evolution of unaltered fluids, allowing for effective application of their derived %VRe formulas across the entire unaltered fluid spectrum, from heavy oil to dry gas. Deviations from indigenous-fluid trends do occur for fluids altered by phase separation, biodegradation, thermal cracking, and thermochemical sulfate reduction (TSR), but corrections can be made to minimize uncertainty in assessing true thermal maturity of altered fluids while respecting other reservoir fluid properties such as gas-to-oil ratio (GOR) and saturation pressure relationships. For instance, although a single charge that has been phase fractionated yields fluids with variable GORs, saturation pressures and slope factors, their butane and pentane isomer ratios remain reflective of the original fluid maturity. In contrast, biodegradation-induced overestimation of maturity based on the isomer ratios of butane and pentane can be corrected by the less affected SF-derived maturity parameter. Reversal to lower apparent SF-derived maturity in thermally and TSR cracked fluids can, on the other hand, be corrected by considering the less affected butane and pentane isomer ratios. Overall, maturities calculated using VLH composition correspond well with fluid type defined based on phase behaviour and source-rock kinetics, thereby putting forward new tools to quantify thermal maturity of reservoir fluids that may be applicable in other petroleum systems.

## Introduction

Engineering classification of petroleum reservoir fluids differs from geochemical classification. Petroleum engineers primarily focus on production-related physical parameters, such as gas-to-oil ratio (GOR), API gravity, viscosity and density measured from in situ representative pressure–volume–temperature (PVT) samples^1^, while geochemists tend to be more concerned with chemical compositions and characteristics, such as isotopes and biomarkers to understand genesis, thermal evolution, migration and subsequent in-reservoir modifications^2,3^. Integration of petroleum engineering parameters and geochemistry can be challenging^4^, but greatly improves reservoir and basin models. This paper discusses the extraction of geochemical parameters, such as the widely used vitrinite reflectance equivalent (%VRe), from preexisting PVT data, and the distinction of original fluid from modified composition. These details can provide higher resolution to fluid mapping and reservoir models, especially when properly interpreted within geochemistry and geology framework.

**Figure 1:**
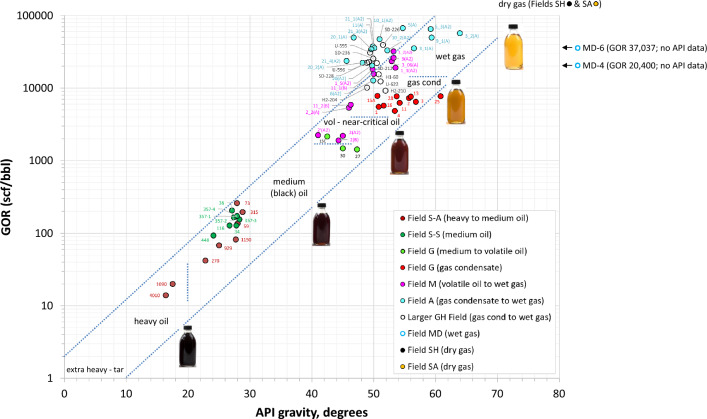
The five main types of petroleum reservoir fluids (black oil, volatile oil, gas condensate, wet gas, and dry gas) plus heavy to extra heavy oils and near-critical fluid, classified according to their API–GOR relationships. Also shown are fluid samples analyzed in this study, ranging from heavy oil to dry gas. Original (or unaltered) fluids have compatible API–GOR relationships (plotting within the blue dotted band), while altered fluids possess incompatible API–GOR relationships.

The five main petroleum fluids identified routinely in reservoir engineering applications include black oil, volatile oil, gas condensate, wet gas, and dry gas^1,5–8^. Other petroleum fluids include heavy oil, extra heavy oil, and near-critical fluid. A practical classification of petroleum fluids according to their API–GOR properties is shown in Fig. 1. In general, a crude that has an API gravity below 22.3° is considered heavy oil, while API gravities < 10° classify crudes as extra heavy oils. A near-critical fluid is light volatile oil or gas condensate with critical temperature and saturation pressure near reservoir temperature and pressure, commonly with a GOR around 3000 standard cubic feet of gas per stock tank barrel of oil (scf/bbl)^8^. Fluid in a trap can be either a direct charge from the kitchen or remigrated from deeper reservoir(s). At regional scale, deeper fields tend to contain gas and lighter fluids that represent direct thermogenic charges from a late-mature source rock, while shallow fields generally contain heavier fluids either from early-mature kitchen nearby or remigrated (or spilled) from deeper traps, according to Gussow’s principles^9^. Exceptions to this include leaky traps that promote leak differential entrapment, leading to lighter fluids in shallower traps due to phase separation^9^.

**Figure 2:**
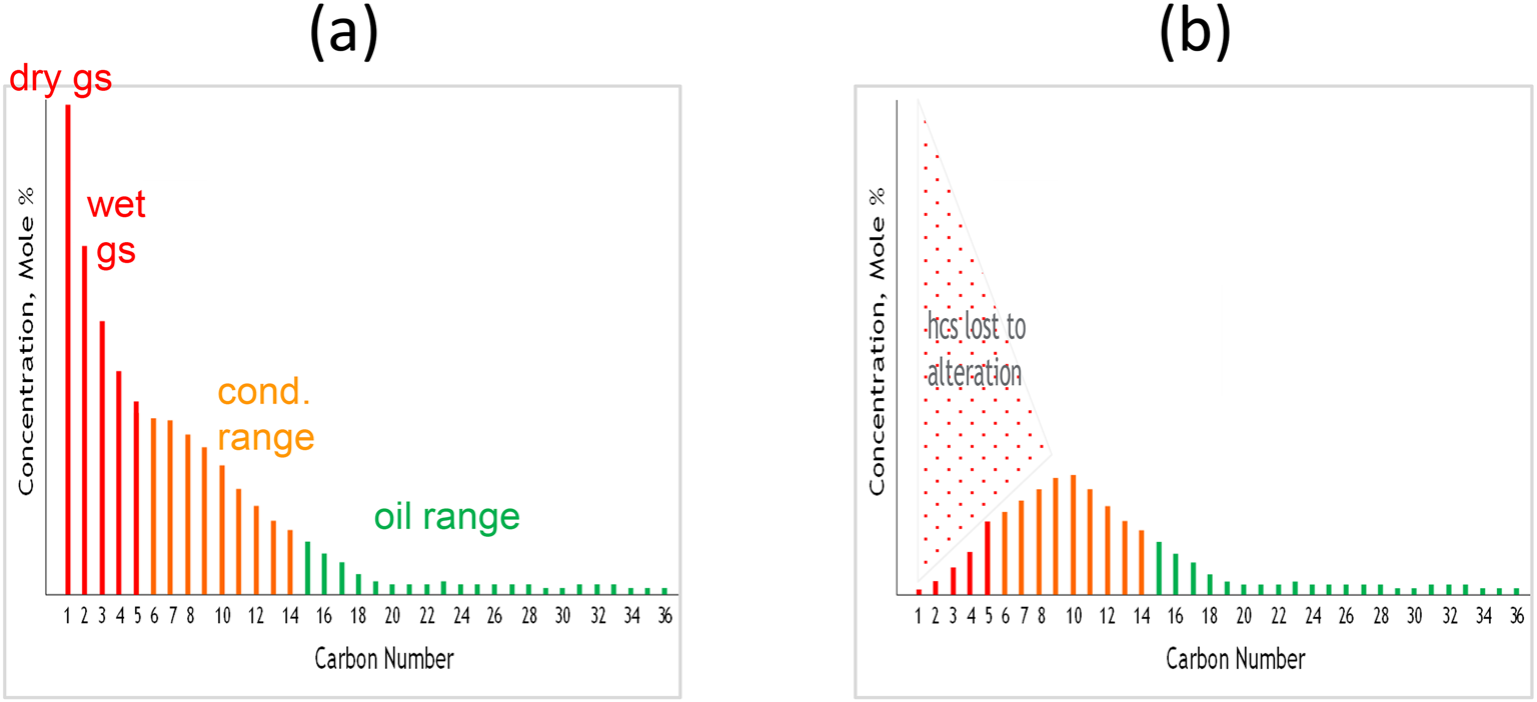
Original (unaltered) fluids have perfect or near-perfect exponential alkane profiles, while altered fluids possess non-exponential compositional distributions.

Compatible API–GOR relationships (Fig. 1) and perfect or near-perfect exponential alkane profiles (Fig. 2) characterise fluids of original (unaltered) composition derived from thermogenic charges, where source maturity is the main control on fluid composition^10^. Altered fluids, on the other hand, are characterised by non-exponential compositional profiles and incompatible API–GOR relationships. Within the reservoir, original fluid composition can be altered by processes like biodegradation, water washing, thermal cracking, thermochemical sulfate reduction (TSR), mixing (recharge), and gas depletion. Loss of gas and volatiles is also not uncommon during sampling and sample preparation. In-reservoir alteration in some cases can be severe, converting a major gas accumulation into a small oil pool^11^, tar or even solid reservoir bitumen^12^. The opposite is also true, where severe thermal cracking at depth can convert an oil accumulation into gas^13^. The state of fluids and the extent of their alteration therefore need to be assessed before any attempt to extract parameters of geochemical significance, such as thermal maturity (vitrinite reflectance equivalent, %VRe), as this greatly impacts proper mapping and prospectivity, as well as petroleum economics, namely, fluid typing, value and recoverability.

Using reservoir fluid PVT data, Thompson^14^ discussed criteria to distinguish original (unaltered/unmodified) gas condensates from hybrid (mixed) accumulations. Unmodified thermal gas condensates are characterised by covariant methane content, GOR, thermal maturity, and saturation pressure (P_sat_). The addition to the reservoir of high-temperature fluids will increase methane, GOR, and pressure regime. However, high-temperature fluids are not the only source for higher GORs, as those can also develop in response to fluid depressurisation that accompanies updip migration, in which case the GOR will inversely relate to P_sat_. A purely thermal unaltered gas condensate necessitates thermal equilibrium between its gaseous and liquid components. This can be validated by similar maturity for both gas and liquid fractions of the same sample using molecular and carbon isotope parameters, many of which were previously discussed based on drill-stem test (DST) samples^15^. Recently, based on reservoir fluid PVT data, Thompson^14^ proposed the slope factor of volatile light hydrocarbons (VLH), namely, propane, n-butane, and n-pentane (C_3_–nC_5_) as a relative maturity parameter, which was shown to covary with stable carbon isotope data. A perfect (R^2^ ≥ 0.999) exponential distribution for the C_2_-C_3_-P_4_-P_5_ suite (where P denotes pseudo-components comprising the respective sum of normal- and iso-alkanes) identifies a pure thermal origin of the VLH^14^. On a logarithmic scale the VLH compositional profiles of unaltered fluids of thermal origin will possess a linear distribution, allowing the slope factor for the molar concentrations of C_3_-nC_5_ to be measured from the exponential Eq. (1)^14^.

1 $$y = A\left( {{e^{ - ax}}} \right)$$

where y = carbon molar concentration; A = intercept; x = carbon number; e^−a^ = slope of the series; slope factor = anti-log of e^a^.

While the gas and liquid fractions in unmixed thermal gas condensates are in thermal equilibrium, hybrid accumulations are distinguishable by greater slope factors (more-mature) in the VLH range compared with those of the less-mature liquid fractions. The slope factor increases with increasing methane and GOR, which in turn result from either a maturity sequence or a phase-separation sequence. A maturity sequence will develop a pressurising system that increases the P_sat_, while depressurisation will lead to a decreasing P_sat_ trend due to phase separation; the differentiation of which can, as discussed above, be achieved by inspecting the GOR–P_sat_ relationship.

**Figure 3:**
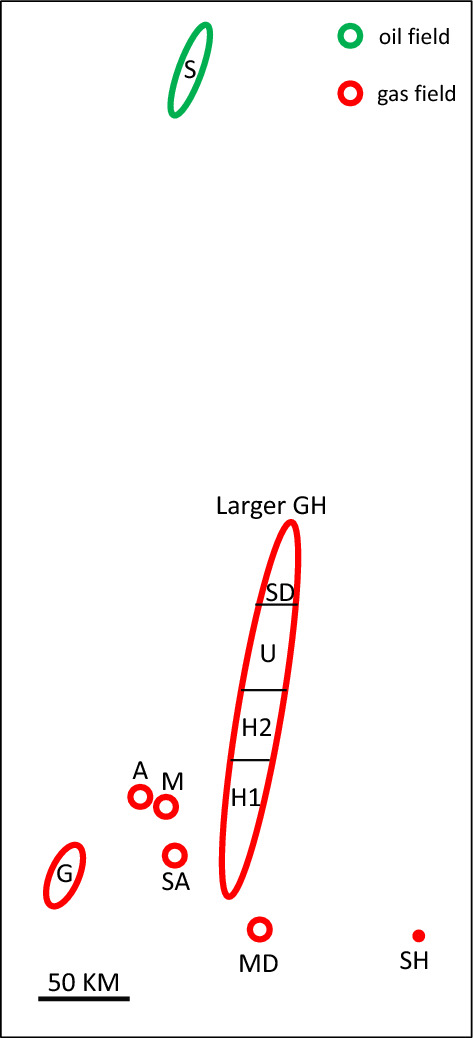
Oil and gas fields sampled for this study, Arabian Peninsula. The oil Field S belongs to the Jurassic carbonate-sourced petroleum system, while all other fields are predominantly gas belonging to the shale-sourced Paleozoic petroleum system.

In the current study, the slope factor of C_3_-nC_5_ volatile light hydrocarbons (abbreviated hereafter as SF) was measured for a set of black to volatile oil and gas condensate samples from a well-constrained field (Field G) where PVT, molecular and stable carbon isotope geochemistry data are predominantly maturity controlled. Fluid geochemistry of this field was previously integrated with fluid inclusions and basin modeling, which inferred indigenous (unaltered) oil and gas condensate accumulations from separate thermal charges^16^. Here, maturity indicators extracted from this field are thoroughly correlated internally while respecting GOR–P_sat_ relationships, with special emphasis on deriving %VRe from the slope factor, iC_4_/nC_4_ and iC_5_/nC_5_ ratios. The applicability of the derived %VRe formulas was then expanded to incorporate fluids from other fields ranging from heavy oils to predominantly gas condensate, wet gas, or dry gas (Fig. 3), which additionally allowed indigenous fluids to be differentiated from fluids that suffered biodegradation, thermal cracking, or TSR.

### Fluid samples and methods

Fluid composition data were collected from in situ representative downhole or recombined wellstream PVT tests from eight fields belonging to two giant petroleum systems (Jurassic and Paleozoic) in the Arabian Peninsula, ranging from heavy to medium oil in the north of the study area to gas condensate, wet gas and dry gas in the south (Fig. 3). Field S contains heavy to medium oil in reservoir A, and medium oil in the underlying reservoir S. Both reservoirs are carbonates of Cretaceous age, and their oil is believed to be sourced from a Jurassic carbonate source rock located to the north. All other (lighter) fluids belong to the Paleozoic petroleum system sourced from a regionally widespread and prolific shale source rock of Early Silurian age, and accumulated either in a Permian siliciclastic reservoir (fields G, M, A, MD, SH, and SA) or an anhydrite-capped carbonate reservoir (the larger GH Field).

Field G is predominantly gas condensate, with black to volatile oil in its southern part. Fluids are believed to be charged via a long-range migration from a kitchen depocenter located to the southeast^16^. Closer to the source depocenter lie fields MD, SH and SA, which contain drier gas, also believed to be sourced from more-mature charges from the same south-lying kitchen depocenter. Further to the north are fields M and A, believed to be sourced from the same Early Silurian shale charging from a smaller kitchen located about 30 km to the northeast of Field M. These fields are predominantly gas condensate to wet gas producers, with volatile oil localised in the southern part of Field M.

Gas condensate (sour gas) samples of variable concentrations of TSR-generated hydrogen sulfide were also included in the current study from the larger GH Field in order to evaluate the effect of TSR on volatile light hydrocarbons. The larger GH Field is broadly subdivided from south to north into four regions (H1, H2, U, and SD). Gas condensates of this field are believed to be predominantly charged from the same Early Silurian shale source rock lying to the east. The Late Permian anhydrite-capped carbonate reservoir fluids are generally H_2_S-free in the shallower south (H1 area), becoming increasingly sour towards the deeper and hotter north, caused by in-reservoir TSR (detailed in Section "Deviation due to TSR alteration").

Geochemical analysis of Field G oil and gas samples included stable carbon isotope analysis (δ^13^C, ^13^C/^12^C, reported in parts-per-thousand, ‰) for the C_1_–C_5_ hydrocarbons using an Agilent 6890 GC equipped with a 30-m Poraplot GSQ column and a Thermo GC Combustion III system interfaced to a Delta Plus stable isotope ratio mass spectrometer (reproducibility ± 0.2‰). The NBS-19 was used as the standard. Aromatic biomarkers were analysed in selected ion monitoring (SIM) mode, monitoring for key ions (m/z 178, 184, 192, 198 and 231), using gas chromatography-mass spectrometry (HP 6890 GC interfaced to an HP mass selective detector MSD 5973). Vitrinite reflectance equivalent (%VRe) was calculated using the phenanthrene and methylphenanthrenes data^17^.

Oil and gas fields represented in figures are labelled with capital letters followed by a numerical suffix referring to different wells. All fields were sampled for one reservoir only except for Field S, which was sampled from two stacked reservoirs identified with an alphabet suffix A and S following the field name, viz*.* S–A and S–S.

### Results and discussion

Field G with oil and gas condensate forms the cornerstone of this paper to study the VLH, given the availability of both PVT and geochemical data and the distinct geochemical characteristics of its fluids that lack any evidence of alteration, before expanding observations to investigate VLH in other fields. The field is predominantly gas condensate, with black to volatile oil discovered in the downdip south at a later stage during field development. The origin of hydrocarbons, charge history, and controls over their distributions were discussed previously using molecular and stable carbon isotope data integrated with fluid inclusions and basin modeling^16^. Briefly, the oil and gas represent thermal charges of variable maturities from the same Early Silurian shale source rock located to the southeast, filling different parts at different times (on average, 150 Ma for the gas condensate, and 120 Ma for the oil)^16^. Here, we interrogate the VLH composition to first demonstrate the pure, unaltered thermogenic origin of this system and subsequently to identify indigenous VLH slope factor, iC_4_/nC_4_ and iC_5_/nC_5_ trends that enable the derivation of valid %VRe values.

#### API–GOR relationship

The oils in Field G range from black to volatile oil according to their GOR (1400–2100 scf/bbl) and API gravity (42.5–47.3°). The GOR for the gas condensates vary from about 4800 to 7800 scf/bbl, with API gravity ranging from 51 to 61°. Compatible API–GOR relationships (Fig. 1) suggest that both fluid types are unaltered thermal fluids derived from two main charging events, as explained below. Other fields used for comparison with Field G, ranging from heavy-medium oil (Field S) all the way to dry gas (fields SH and SA), are also plotted on Fig. 1.

#### Slope factor in relation to methane content, GOR, and saturation pressure

**Figure 4:**
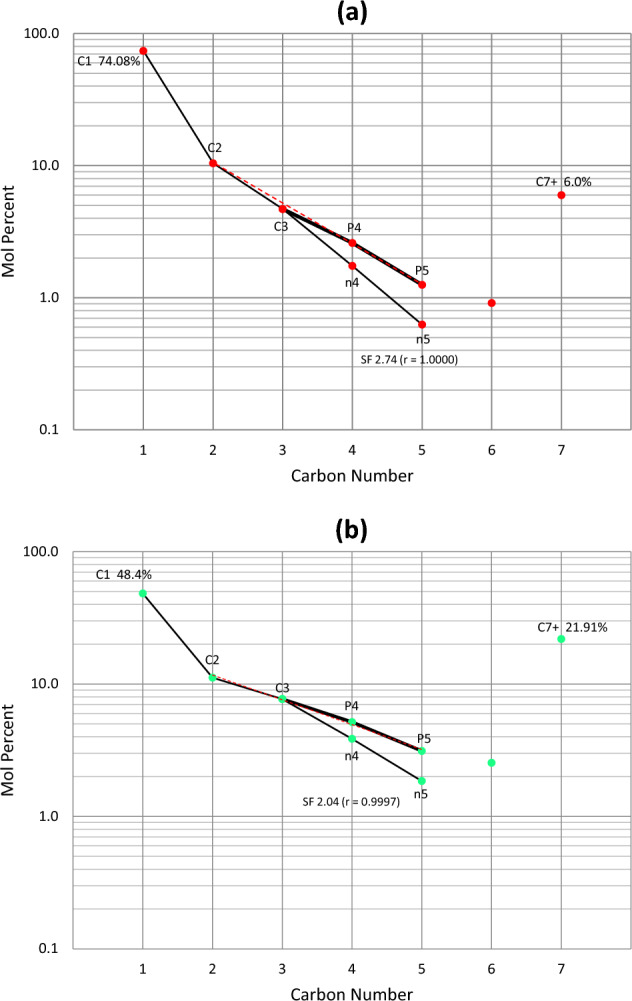
Compositional profiles for (**a**) gas condensate from well-2, and (**b**) volatile oil from well-30, showing representative slope factors for the C_3_–nC_5_ range, abbreviated SF. The red dashed line represents the predicted exponential C_2_–C_3_–P_4_–P_5_ progression, which is only slightly different to the observed distribution, consistent with pure thermal origin for both oil and gas condensate. This, together with perfect fit for the C_3_–nC_4_–nC_5_, provide accurate slope factors.

Both sets of oil and gas condensate samples of Field G have perfect or near-perfect alkane profiles preserving exponential C_2_–C_3_–P_4_–P_5_ relationships (as compared with the predicted progression marked by the red dashed line)—evidence for intact fluids from original charges with no in-reservoir modifications. Slope factors for the fluid samples were measured from the exponential Eq. (1)^14^. Compositional profiles for a gas condensate (well-2) and a black oil (well-30) are shown in Fig. 4 as examples, together with their measured SF values of 2.74 and 2.04, respectively.

**Figure 5:**
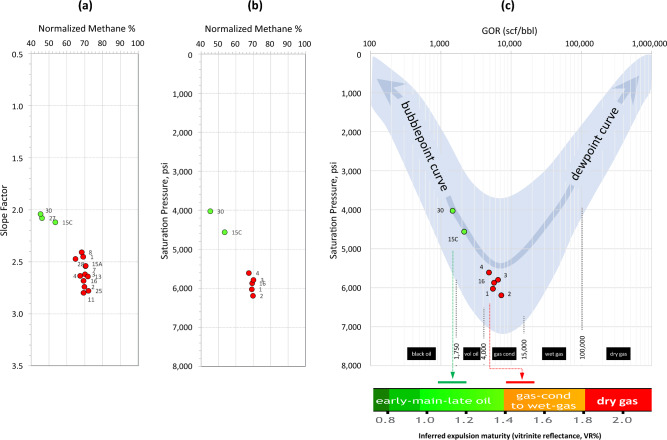
Relationships of (**a**) slope factor with normalized methane content, (**b**) saturation pressure with normalized methane content, and (**c**) saturation pressure with GOR for oils (green) and gas condensates (red) of Field G. The blue band in panel c represents the global GOR–P_sat_ trend^18,19^. Methane normalized to hydrocarbon content only (C_1_/C_1-7+_).

Interrelationships between measured SFs, methane content, and saturation pressure data for Field G fluids are illustrated in Fig. 5. The SF increases with the increase in methane, which is associated with an increase in saturation pressure and GOR. This covariance indicates that the gas condensates were derived from a separate, more-mature charge than the oil, rather than phase separation of a single charge. Phase separation accompanying updip migration implies depressurisation and—despite resulting in higher methane and GOR—would lead to a decrease in saturation pressure along the dewpoint pressure trend indicated in Fig. 5c, which is not the case for Field G fluids.

#### Comparison of slope factor with δ^13^C, %VRe, and C_4_ and C_5_ isomer ratios

**Figure 6:**
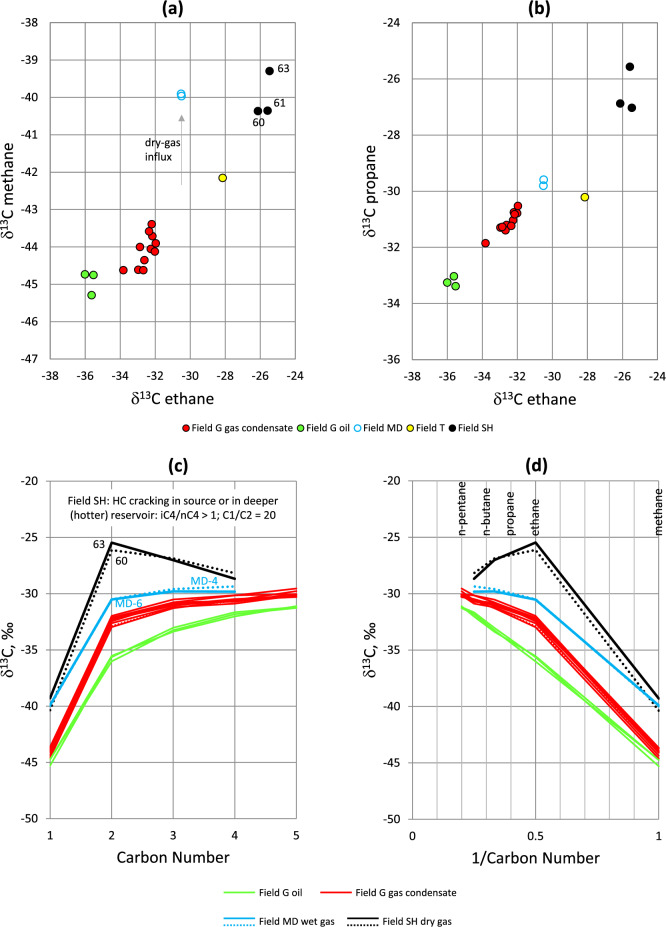
(**a**) δ^13^C ethane versus δ^13^C methane for Field G oils and gas condensates, in comparison with drier gas fields located to the southeast. (**b**) δ^13^C ethane versus δ^13^C propane for Field G oils and gas condensates, in comparison with drier gas fields located to the southeast. (**c**) δ^13^C fingerprinting of the gas range in both the oils and gas condensates of Field G, wet gas in Field MD and dry (cracked) gas in Field SH. (**d**) Chung natural gas plot.

Within regional framework, Field G fluids represent early expulsion products of a larger migration system charging from a southeasterly kitchen^16^. Gas in areas located to the southeast of Field G, such as fields MD, T and SH (Fig. 6), becomes progressively drier and isotopically heavier (δ^13^C less negative) towards the kitchen depocenter, as represented by the δ^13^C crossplots for methane, ethane and propane (Fig. 6a,b), suggesting that Field G fluids represent earlier, less-mature charge events. Within Field G, isotope maturity differences between the less-mature solution gas of the oils and the more-mature gas condensates persist. This is further evidenced on δ^13^C fingerprinting for the C_1_–C_5_ gas range (Fig. 6c). Gases from a common source generate normal isotopic distributions that show progressive ^13^C enrichment with increasing carbon number, where δ^13^C_1_ < δ^13^C_2_ < δ^13^C_3_ < δ^13^C_4_ < δ^13^C_5_ (e.g., refs. ^20,21^). This is expressed on Chung natural gas plot by a linear or sublinear trend between carbon isotopic ratios and the inverse carbon numbers^22^ (Fig. 6d). Both fluid types in Field G show normal isotope profiles that are consistently lighter (more negative) for the oil’s solution gas compared with gas condensates, suggesting that (1) the solution gas was cogenerated with oil at relatively lower maturity than the gas condensates, and (2) in each fluid system, the full gas range (e.g., C_1_–C_2_ vs. C_4_–C_5_) was derived from the same source.

Carbon isotope data agree with the observed covariance of the slope factor and methane content, GOR, and saturation pressure discussed above, collectively indicating a reservoir pressurization system controlled primarily by charge maturity, rather than phase separation of the same charge. Adding more-mature fluids from a source rock of higher temperatures increases the SF, methane, GOR and P_sat_. Such a well-constrained thermal system validates the use of the slope factor as a maturity indicator and possibly also as a rough guide for fluid phase (GOR) and P_sat_.

**Figure 7:**
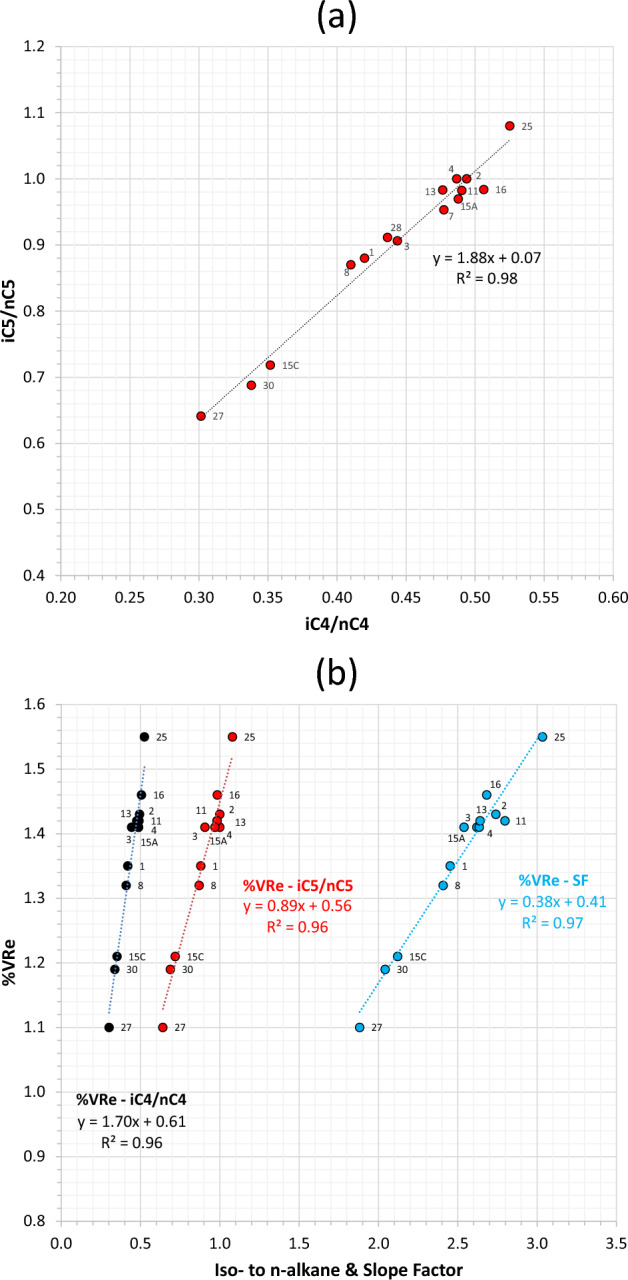
(**a**) Intercorrelation between iso- to n-alkane ratios at C_4_ and C_5_ for oil and gas condensate samples. (**b**) %VRe correlation with iC_4_/nC_4_, iC_5_/nC_5_ and slope factor.

In addition to slope factor, the isomer ratios for C_4_ and C_5_ also provide excellent means for assessing relative maturity for both conventional and unconventional petroleum systems^23–28^. For Field G fluids, the isobutane/n-butane (iC_4_/nC_4_) and isopentane/n-pentane (iC_5_/nC_5_) ratios correlate positively very well (R^2^ = 0.98, Fig. 7a). The iC_4_/nC_4_ increases from 0.3–0.35 for oils to 0.41–0.53 for gas condensates, while the iC_5_/nC_5_ ranges from 0.64–0.72 for oils to 0.87–1.08 for gas condensates. Most thermogenic gases have an average iC_4_/nC_4_ ratio of 0.5 (ref.^27^). The two ratios are in excellent positive correlation (R^2^ = 0.96, Fig. 7b) with vitrinite reflectance equivalent (%VRe) derived from the methylphenanthrene index^17^ measured on the same sample set, which in turn shows a strong positive correlation (R^2^ = 0.97) with slope factor. A strong positive correlation between the iC_4_/nC_4_ ratios from natural gas samples and vitrinite reflectance measured on related drill-core samples were also reported from the tight-gas system of the Montney Formation in western Canada^28^.

The consistency of maturity for the gaseous and liquid components in each fluid type of Field G (collectively manifested in SF, iC_4_/nC_4_, iC_5_/nC_5_, δ^13^C, GOR, P_sat_, and %VRe) indicates that (1) the gas from the oil wells is an oil-associated gas generated at lower maturities than that for the gas condensates, (2) the gas condensates are thermal in origin, as opposed to phase separation or evaporative fractionation, and (3) there is no mixing between the two fluid systems. Given the excellent correlation between the various PVT fluid composition data and geochemical data that lack any evidence of alteration, it was possible to derive the following formulas to quantify %VRe using the slope factor and the butane and pentane isomer ratios for indigenous fluids (Fig. 7b; Eqs. 2, 3, and 4, below).

2 $$\% VRe\left( {SF} \right) = 0.38 \, SF + 0.41; \, {R^2} = 0.97$$

3 $$\% VRe\left( {i4} \right) = 1.70 \left( {i{C_4}/n{C_4}} \right) + 0.61; \, {R^2} = 0.96$$

4 $$\% VRe\left( {i5} \right) = 0.89 \left( {i{C_5}/n{C_5}} \right) + 0.56; \, {R^2} = 0.96$$

### Validation and application in other oil and gas fields (heavy oil to dry gas)

#### VLH compositional profiles

**Figure 8:**
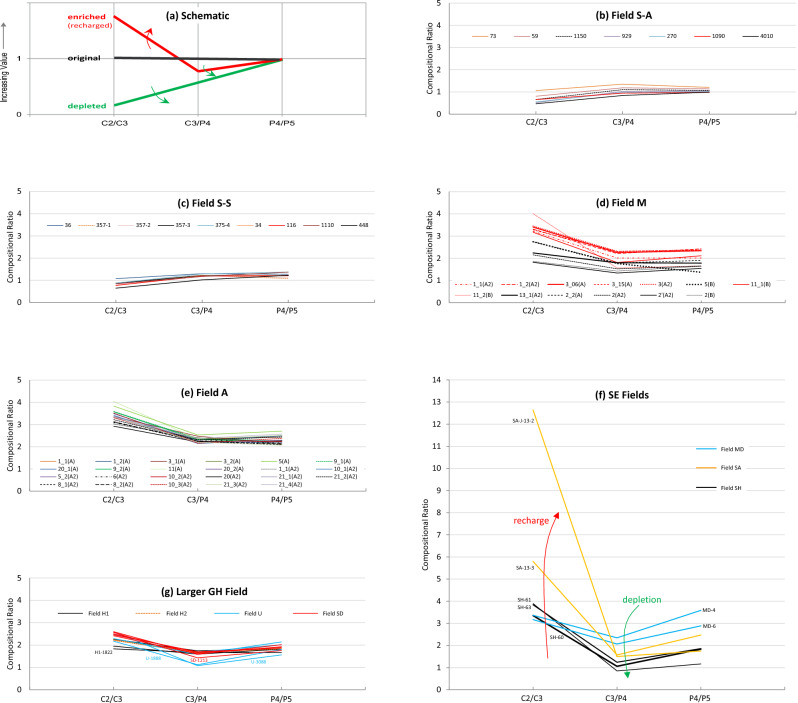
(**a**) Schematic compositional profiles of C_2_/C_3_, C_3_/P_4_ and P_4_/P_5_ ratios to assess the nature of fluids (original, depleted, or enriched), which reflect charge and post-charge history, (**b**–**g**) actual compositional profiles for various oil and gas fields selected for comparison with Field G. Each profile represents a well. Some wells have multiple PVT tests indicated by the test number following the well number, where applicable.

VLH inter-compound relationships in fields with fluids heavier and lighter than those in Field G were analyzed in order to study the behaviour of slope factor in different types of fluids ranging from heavy oil to dry gas. Again here, before assessing the behaviour of slope factor in relation to methane content, GOR and saturation pressure, the state of VLH (i.e. original versus altered) needs to be assessed. As illustrated by Thompson^14^, this can be achieved by inspecting the compositional profiles for the C_2_/C_3_, C_3_/P_4_ and P_4_/P_5_ ratios, with the concept illustrated schematically in Fig. 8a. Upon thermal generation and expulsion from the source rock, the VLH concentrations form an exponential series, yielding equal C_2_/C_3_, C_3_/P_4_ and P_4_/P_5_ ratios^14^. Exponential series of VLH concentrations were also previously reported in oils and gas condensates^29^ and experimentally by pyrolyzing petroleum asphaltenes to model oil evolution^30^. Fluids that suffered loss or depletion of lighter gases will be characterised by a C_2_/C_3_ < C_3_/P_4_ < P_4_/P_5_ relationship, while fluids enriched by a later influx of drier gas (mainly methane and ethane) will possess the opposite trend (C_2_/C_3_ > C_3_/P_4_ > P_4_/P_5_). A minimum at C_3_/P_4_ signifies depletion followed by recharge.

**Figure 9:**
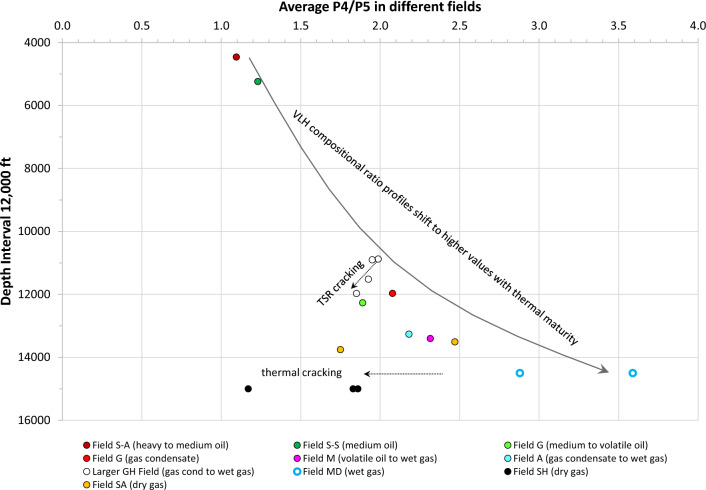
Evolution of the P_4_/P_5_ ratio with thermal maturity of fluids from two major petroleum systems. Being the least affected by alteration, the P_4_/P_5_ ratio is chosen to represent the evolution of the entire VLH compositional profiles C_2_/C_3_, C_3_/P_4_, and P_4_/P_5_ ratios. The shallow Field S represents Jurassic carbonate-derived fluids from the Jurassic petroleum system, while the deeper fields represent Paleozoic shale-derived fluid systems.

The P_4_/P_5_ ratio is usually the least affected by alteration. Using the averaged P_4_/P_5_ ratio in each field as a reference to represent the entire VLH compositional profile (linking C_2_/C_3_–C_3_/P_4_–P_4_/P_5_ ratios), the regional evolution of these profiles with maturity can be assessed when plotted against depth (Fig. 9). The ratio ranges from 1.1 in the shallowest oil field, increasing steadily with depth to as high as 3.6 in the gas fields. Although the shallow oil and deep gas fields were derived from different source-rock lithologies of different ages (Jurassic carbonate compared with Paleozoic shale), maturity (rather than source variations) appears to be the controlling factor over the shift of compositional profiles to higher values. Progression of compositional profiles to higher values with increasing depth and maturity is predictable given the higher concentrations of VLH in the deeper, more-mature fields. A shift or reversal to lower P_4_/P_5_ ratio (and hence the overall compositional profile) is, however, noticed in dry gas fields SH and SA (Fig. 9), apparently due to thermal cracking, and in the larger GH Field due to TSR cracking. Effects of thermal evolution and alteration processes on the VLH inter-compound relationships and their use for maturity estimations are discussed in subsequent sections.

**Figure 10:**
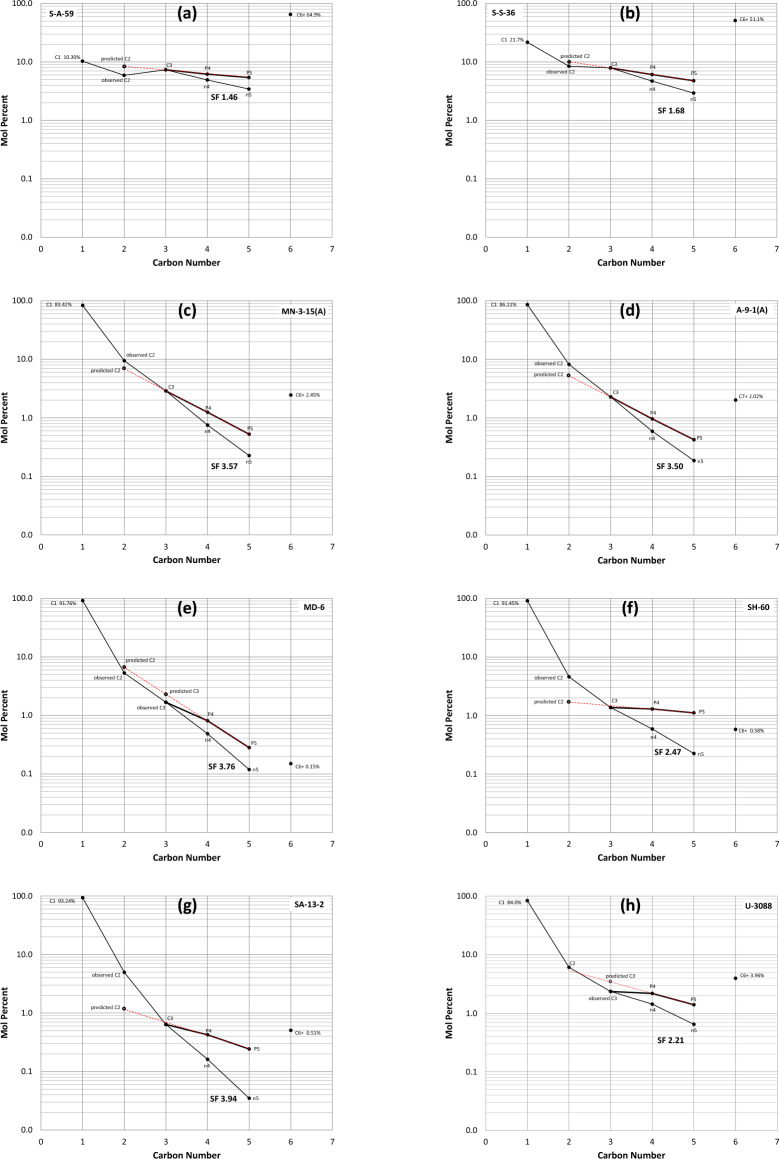
Compositional concentration profiles representative of the different fields. Predicted C_2_ represents the extension of the exponential C_2_–C_3_–P_4_–P_5_ progression. *SF* slope factor of C_3_-nC_5_ suite.

More interestingly, different fields are characterised by different compositional ratio profiles, suggesting variable charge-alteration histories (Fig. 8b–g). The heavy to medium oils of the shallow Field S have equal or near-equal C_3_/P_4_ and P_4_/P_5_ ratios, with only a slight drop in the C_2_/C_3_ ratio (Fig. 8b,c), suggestive of a minor loss of ethane (and expectedly methane) either during migration or within the reservoir. The minor loss of methane-ethane did not alter the generative C_3_–P_4_–P_5_ series, which renders the slope factor measured valid (examples showing SF 1.46 and 1.68, Fig. 10a,b). In contrast, no depletion is evident in the deep Palaeozoic volatile oils, gas condensates and wet gas in Field M and the neighbouring Field A. These fields are also characterised by equal or near-equal C_3_/P_4_ and P_4_/P_5_ ratios, consistent with a thermal origin, but with an additional drier gas charge evidenced by elevated C_2_/C_3_ ratios (Figs. 8d,e, 10c,d). Note that the volatile oils in the southern part of Field M (samples shown in black in Fig. 8d) have apparently experienced the smallest recharge, being displaced and largely locked from the drier north of the field.

Drier gas discoveries in fields MD, SH and SA are closer to the south-lying kitchen depocenter than Field G and are therefore expected to have been subjected to multiple fill-spill cycles ending with drier fluids. Interrelationships of the VLH suites in these fields indeed suggest complex fluid histories that involve partial depletion followed by drier gas recharge (Fig. 8f). Depletion is indicated in the ratio profiles by a minimum at C_3_/P_4_, with recharge bouncing the C_2_/C_3_ ratio back to higher levels. In the concentration profiles (Fig. 10e–g), depletion is hinted by a reduction in C_3_ (predictably also in C_1_ and C_2_), while the dry-gas recharge is indicated by enriched C_2_ accompanied by very high methane > 91%. Despite the dry gas recharge at Field MD, the observed C_2_ and C_3_ concentrations remained below what may be predicted based on the C_3_-P_4_-P_5_ progression trend, suggesting that the recharge was very dry and more mature than preexisting C_2+_ fluids. This inference agrees with the carbon isotope discrepancy between the ethane and the isotopically less-negative (more-mature) methane (Fig. 6a).

A special case of hydrocarbon depletion caused by TSR destruction is represented here by the larger GH Field that dips to the north. Fluids in the shallowest region (H1) in the south show almost flat compositional ratio profiles and become increasingly depleted towards the deeper north (regions U and SD), as indicated by a minimum at C_3_/P_4_ (Fig. 8g). Depletion caused by the consumption of hydrocarbons via TSR leads to the formation of H_2_S. A representative compositional concentration profile for a strongly TSR-altered fluid sample (U-3088, 29% H_2_S) is shown in Fig. 10h, exhibiting reduced propane concentrations, thereby altering the generative C_3_-P_4_-P_5_ series, which caused shifts to lower SF values away from the generative SF trend without being affected by the more recent methane- and ethane-rich charge represented by higher C_2_/C_3_ ratios (Fig. 8g). A detailed account on TSR and its control on compositional alteration in this field is handled in Section "Deviation due to TSR alteration".

#### Indigenous slope factor and iC_4_/nC_4_ trends and effect of alteration processes

**Figure 11:**
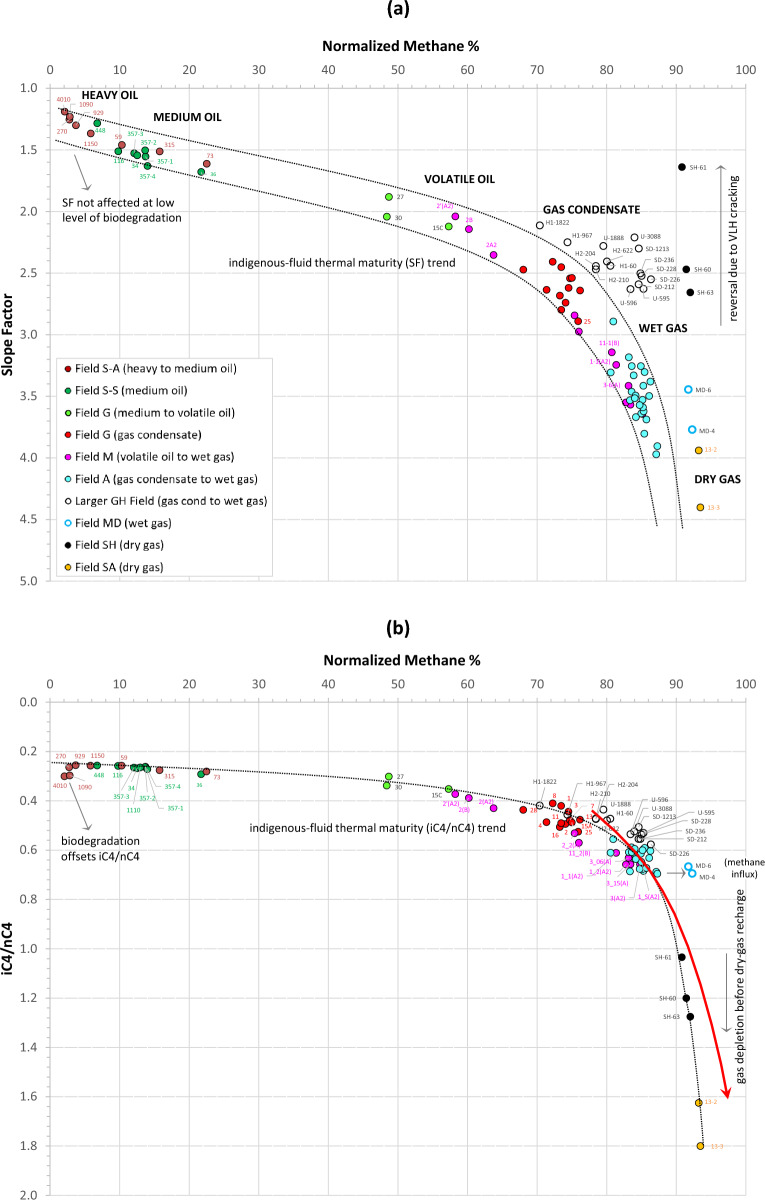
Relationship of normalized methane content with (**a**) slope factor and (**b**) iC_4_/nC_4_ ratio. The red arrow represents the indigenous thermal maturity trend observed in the tight-gas system of the Montney Formation in western Canada^28^. Methane normalized to hydrocarbon content only (C_1_/C_1-7+_).

Unaltered thermogenic fluids with equal or near-equal C_3_/P_4_ and P_4_/P_5_ ratios are expected to yield slope factors that accurately represent expulsion maturity levels, with compatible methane and GOR relationships. A sign for the unaltered state of the majority of fluids tested here (from heavy oils in Field S all the way to wet gas in fields M and A) is their slope factors that increase systematically with the increase in methane, forming an indigenous-fluid SF trend (Fig. 11a), expanding the trend discussed above for unaltered Field G oils and gas condensates. Variable degrees of deviation from the indigenous slope factor and iC_4_/nC_4_ trends are observed in fluids suffered biodegradation, thermal cracking, or TSR, as detailed below.

#### Deviation due to biodegradation

Thermal maturity increases the iC_4_/nC_4_ ratio^24,27,28^. The ratio, however, is also sensitive to biodegradation because n-butane biodegrades at a faster rate than isobutane, thereby increasing the ratio^31,32^. Oil field S-A is the shallowest field examined in the current study and the only field with evidence for biodegradation (in some samples). As shown in Fig. 1, the oil here grades from medium to heavy, with sample 73 at the structural crest recording the highest GOR and API gravity, both steadily decreasing towards the oil–water contact (samples 4010 and 1090). This bulk density gradient is mirrored in an overall decrease with depth in methane and slope factor (Fig. 11a), suggesting a combined density segregation and maturity stratification effect. Despite being the heaviest and least-mature, the deepest samples in this oil column (4010 and 1090) show slightly higher-than expected iC_4_/nC_4_ values, apparently due to biodegradation, thereby offsetting from the indigenous-fluid trend (Fig. 11b). Maturity in this case needs to be verified by the slope factor, which appears to provide a more accurate estimate of thermal maturity in biodegraded reservoirs (Fig. 11a).

#### Deviation due to thermal cracking

A small deviation from the indigenous slope factor trend is observed for Field MD wet gas. A larger disparity is exhibited by the dry gas in fields SH and SA (Fig. 11a). Despite the slight drop in C_2_ and C_3_ and the shift to higher methane content from a very-dry-gas recharge, the slope factor for the wet gas in Field MD remained largely unaffected and usable, exhibiting values comparable to other wet gas samples from fields M and A (Fig. 11a). The slope factor in this case represents the minimum thermal maturity of total fluid. Gas isotope profiles of Field MD are less negative than Field G gas condensate isotope profiles (Fig. 6c,d), consistent with their higher maturity (wet-gas state), which is also in agreement with a maximum iC_4_/nC_4_ ratio of 0.69 for Field MD compared with 0.53 for Field G gas condensates. Most thermogenic gases have an average iC_4_/nC_4_ ratio of 0.5 (ref.^27^) while higher ratios usually signify higher expulsion maturities^24,27,28^ or thermal cracking^33^, although, as explained in Section "Deviation due to biodegradation", biodegradation also results in abnormality in the iC_4_/nC_4_ ratio^31,33^. In shale-gas systems, the iC_4_/nC_4_ (and iC_5_/nC_5_) ratio initially increases due to generation of gas directly from kerogen, but is rolled over once oil cracking starts, and is completely reversed upon wet-gas cracking, due to higher cracking rates of isobutane and isopentane relative to their normal alkane counterparts in closed (unconventional) systems^25,26^.

The dry gas in fields SH and SA that suffered variable degrees of depletion before drier-gas recharge (as discussed in Section "VLH compositional profiles") exhibits shifts to higher methane content and relatively reduced slope factors (Fig. 11a). A clue behind the SF reversal can be gained by inspecting corresponding iC_4_/nC_4_ ratios, which are shown to be higher than unity in both fields (Fig. 11b), suggesting hydrocarbon migration from hotter/deeper reservoirs where thermal cracking occurred. Thermal cracking favours the formation of isobutane and isopentane at the expense of their normal alkane counterparts^26,33,34^. Rollover and reversal caused respectively by oil and wet-gas cracking (where iC_4_/nC_4_ ratio decreases with increasing methane at high thermal maturity > 95% methane in the late-gas window) are frequently reported in unconventional (closed) gas systems, such as the Barnett Shale in Texas^25,26^. The rollover/reversal is not observed in the conventional reservoirs of the current study (Fig. 11a), as well as in many other petroleum systems, for example, the tight-gas system of the Montney Formation in western Canada^28^ and coal-derived gases of the Triassic Xujiahe Formation in central Sichuan basin, even at very high iC_4_/nC_4_ and iC_5_/nC_5_ ratios of up to 1.76 and 3.0 respectively^35^. An offset to methane contents higher than the indigenous trend at a given iC_4_/nC_4_ maturity is interpreted for the Montney gas system to represent ‘excess methane’ enriched by uplift-induced phase separation followed by selective migration (or transmission) of methane through coarser-grained siltstone bands or fractures^28^.

**Figure 12:**
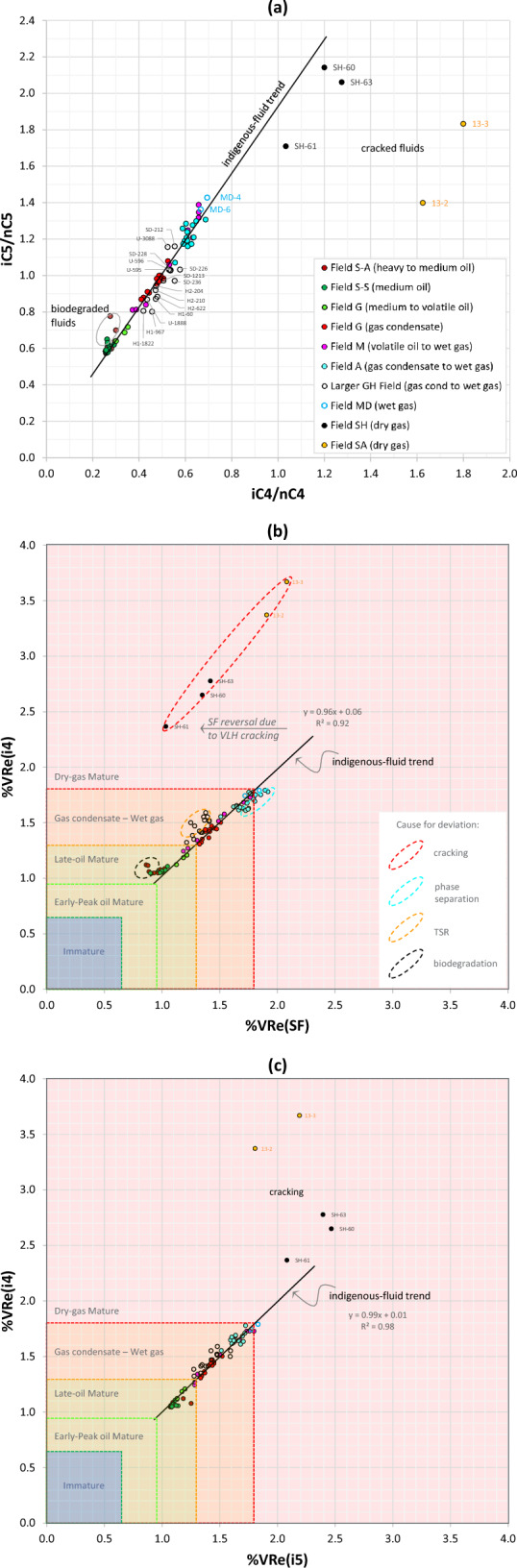
Crossplots of (**a**) iC_4_/nC_4_–iC_5_/nC_5_, (**b**) %VRe(SF)–%VRe(i4), and (**c**) %VRe(i5)–%VRe(i4).

Thermal cracking, however, appears to smooth out the C_3_-P_4_-P_5_ trend, hence reducing (or reversing) the slope factor, most evident in Field SH (Figs. 10f and 11a). The SF reversal in this field coincides with a deviation (or reversal) in the carbon isotope profile, in which the ethane is isotopically heavier than propane, which in turn is heavier than butane (Fig. 6c,d), apparently due to thermal cracking of wet gas (C_3+_) components in a deeper/hotter reservoir. As such, the iC_4_/nC_4_ versus methane plot (Fig. 11b) and iC_4_/nC_4_ versus iC_5_/nC_5_ plot (Fig. 12a) are particularly useful to check against the accuracy of slope factor in wet- to dry-gas fields where contributions from cracked hydrocarbons are suspected. Why thermal cracking makes iC_4_/nC_4_ increasing faster than iC_5_/nC_5_ here (Fig. 12a) is not totally clear. A likely explanation is that n-butane cracks faster than n-pentane, hence increasing the iC_4_/nC_4_ at a faster rate than iC_5_/nC_5_. It is also possible that the reduction in the iC_5_/nC_5_ ratio is due to mixing with contributions from gas cracked in the source rock itself known to be overmature in much of the depocenter.

#### Deviation due to TSR alteration

**Figure 13:**
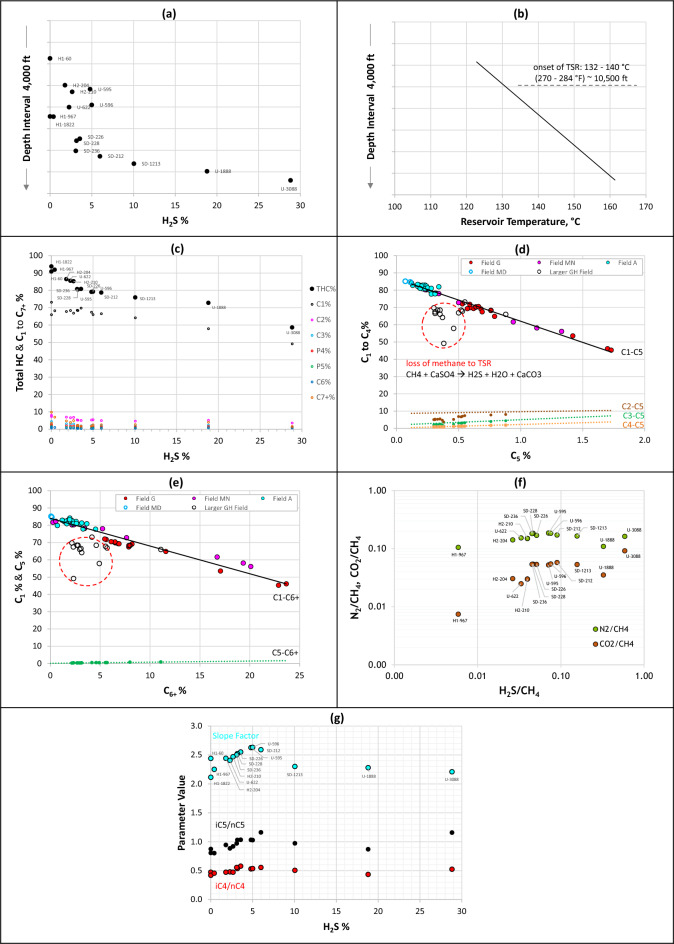
Crossplots for the larger GH Field gas condensates: (**a**) Depth profile of TSR-generated H_2_S%, (**b**) reservoir temperature profile, (**c**) relationships between H_2_S concentrations and total hydrocarbons and C_1_ to C_7+_ percentages, (**d**) pentane versus methane to butane percentages showing much greater loss of methane to TSR relative to other gases when compared with the generative trend of fluids unaltered by TSR, (**e**) C_6+_ versus C_1_ and C_5_ percentages, (**f**) positive correlations among the non-hydrocarbon gas components suggestive of their control by TSR, (**g**) impact of TSR on maturity parameters: slope factors, iC_4_/nC_4_, and iC_5_/nC_5_ ratios.

Deviation from the indigenous SF-methane trend is also observed for sour gas from the larger GH Field (Fig. 11a). The north–south-trending field is subdivided into four regions (H1, H2, U, and SD) that, in this reservoir, generally become increasingly enriched in hydrogen sulfide with increasing depth and temperature to the north (Fig. 13a,b). Fieldwide present-day reservoir temperature profile is shown in Fig. 13b. The reservoir is sitting at its maximum paleotemperature. The carbonate reservoir in question is capped by (and variably intercalated with) anhydrite. Gas souring in this and equivalent reservoirs regionally are controlled by in-reservoir TSR of anhydrite that generally intensifies with increasing reservoir depth and temperature^36^. Various TSR temperature thresholds have been reported from different geological settings worldwide (ref.^37^ and refs therein). In the larger GH Field, 132–140 °C (270–284 °F) appears to represent the temperature threshold for TSR and subsequent H_2_S formation, reaching about 29 mol% in the current set of samples from the deepest U region (Fig. 13a). In addition to temperature, other factors control the threshold and intensity of TSR, which may explain infrequent occurrences of TSR-related H_2_S above and below the threshold temperature identified here. These include the thickness and porosity of the reservoir and the distribution and nature of anhydrite that varies from finely crystalline nodules to massive layers^36^, among other factors^38–40^. Exceptions (not shown here) do indeed occur, where certain pockets below the depth-temperature thresholds are sweet, while some fluids above are sour. Lack of H_2_S at depth could be due to lack of reactive anhydrite, loss via leakage, scrubbing by clay minerals, or recent charge of sweet gas. Sour fluids above the depth and temperature thresholds could represent remigrated fluids from deeper, previously altered, accumulations, or due to the presence of reactive finely grained anhydrite nodules and intraformational seals known to occur locally within the reservoir.

This section focuses on the role of TSR in altering the volatile light hydrocarbon composition, in particular the three maturity parameters: slope factor, iC_4_/nC_4_ and iC_5_/nC_5_. Figure 13 presents the depth and temperature profiles of TSR-generated H_2_S, together with the impact on VLH distributions and on related maturity parameters. TSR involves the in situ formation of H_2_S at the expense of hydrocarbons and the conversion of anhydrite to calcite, following the general formula CaSO_4_ (anhydrite) + CH_4_ → CaCO_3_ (calcite) + H_2_S + H_2_O, assuming methane as the primary hydrocarbon involved. This explains the observed reverse relationship between H_2_S and total hydrocarbon and especially methane contents (Fig. 13c).

TSR preferentially attacks liquid hydrocarbons before attacking the most resistant methane, the latter occurring at temperatures above 140 °C^36^ and possibly as high as 200 °C^37^. TSR oxidation can also be methane-dominated in drier gas fields^42^. In the larger GH gas condensate field, even the methane appears to be affected by TSR. The increase from no H_2_S to approximately 29% in the most-TSR-affected sample corresponds to approximately 29% drop in methane content (from ~ 70% to ~ 50%), with apparently only minor contributions from C_2+_, mainly ethane (Fig. 13c), suggesting that the TSR here is methane-dominated. Given that methane is the most thermally stable compound among other hydrocarbons^43,44^, it is expected that the TSR oxidation of methane was preceded by the oxidation of the C_2+_ range. Observed C_2+_ contents therefore likely represent mixing with a later low-methane wet gas charge. This is illustrated when comparing alkane trends for this field with the generative trends of non-TSR-altered (H_2_S-free) fields (Fig. 13d,e), showing the greatest apparent loss to TSR occurring for methane with respect to other components replenished by the wet-gas recharge, represented in Fig. 13d,e by n-pentane and hexane-plus, respectively. As indicated by Jenden et al.^45^, the oxidation of methane in the region is indicated by (1) covariance of H_2_S/CH_4_ with CO_2_/CH_4_ and N_2_/CH_4_ (as manifested for the current samples in Fig. 13f), (2) significant enrichment in ^13^C (δ^13^C from as low as − 40‰ to as high as − 3‰), and (3) abundant calcite cement rimming fine-grained anhydrite nodules, together with occasional pyrite, sphalerite and galena—collectively a reflection of TSR control on methane and non-hydrocarbon abundances. Base metal sulfide mineralization consumes hydrogen sulfide; hence, the reported occasional presence of such minerals in this reservoir suggests that the original H_2_S/CH_4_ ratio was once significantly higher^45^. Using methane δ^13^C and N_2_/CH_4_ relationships to model the effects of TSR on the chemical and stable isotope composition of a dry gas, it was estimated that more than ¾ of the original methane charge has been destroyed by TSR^45^. As discussed in Cai et al.^42^, water is expected to be generated in methane-dominated TSR system. Evidence for water generation in the larger GH sour-gas accumulation includes the decrease in both water salinity of fluid inclusions and δ^18^O values of associated calcite^46^.

Relative reactivity of the most reactive ingredients, the saturated hydrocarbons, during TSR was recently discussed and concluded to be not fully clear yet^47^. It was tentatively proposed that iso-alkanes are preferentially lost to TSR compared with normal alkanes, causing ratios like pristane/nC_17_, phytane/nC_18_, and iC_4_/nC_4_ to decrease as TSR intensifies, with variable oxidation tendencies for the C_2_-C_4_ range at later stages of TSR. Similar petroleum systems where methane and ethane were suggested to be involved in TSR include the Triassic Wolonghe field in the Sichuan basin^48^ and the Feixianguan Formation in East Sichuan basin in China^49^, where it is indicated to be methane-dominated in the latter.

The current study of gas condensate-wet gas in the larger GH field, focusing on deriving VLH-based maturity parameters in TSR-altered accumulations, therefore offers insights on the behaviour of VLH during TSR, where the biggest loss to TSR is seen for methane followed by ethane, with only minor impact on C_3+_ (Fig. 13d). Subsequently, the TSR impact on VLH-derived maturity parameters at low H_2_S concentrations is also negligible (Fig. 13g). The slope factor appears insignificantly affected up to 5% H_2_S, while the accuracy of iC_4_/nC_4_ and iC_5_/nC_5_ ratios appears to persist to slightly higher H_2_S levels (6–8%), above which an infliction occurs in all three maturity parameters. An exception is noticed for the most TSR-altered sample U-3088 with ~ 29% H_2_S, showing a return to higher iC_4_/nC_4_ and iC_5_/nC_5_ values (but not for the slope factor, Fig. 13g). This introduces a certain level of uncertainty when assessing the thermal maturity of TSR-affected accumulations using VLH range, especially where H_2_S > 8–9%. Figure 10h shows the compositional concentration profile for the most-TSR-altered fluid (U-3088, 29% H_2_S) exhibiting a reduced propane concentration, while the butane and pentane are apparently not or less affected, thereby altering the generative C_3_–P_4_–P_5_ series (Fig. 10h) and causing the shift of slope factor to values lower than expected based on the generative slope factor trend (Fig. 11a). TSR alteration was followed by a more recent methane- and ethane-rich charge documented in the compositional ratio profiles by a V shape (Fig. 8g), and in the compositional concentration profiles (Fig. 10h) by higher-than-predicted methane and ethane concentrations. This supports speculations discussed above that the original H_2_S/CH_4_ was once significantly higher before recharge with fresh unaltered gas.

In comparison with the slope factor, the iC_4_/nC_4_ ratio behaves more systematically with variations in methane content (Fig. 11b) and with the less TSR-affected iC_5_/nC_5_ counterpart, both exhibiting only minimal scatter from the indigenous (non-TSR-altered)-fluid trend (Fig. 12a). The scatter within the iC_4_/nC_4_ and iC_5_/nC_5_ ratios for the TSR-altered field is within the scatter observed for non-TSR-altered samples (Fig. 12a), allowing for their use for relative maturity estimation and the derivation of more quantitative maturity parameters, such as %VRe. Consideration of all three maturity ratios is important in order to provide more accurate maturity estimations, especially in altered petroleum samples, while respecting saturation pressure data, as discussed below. 

##### %VRe(SF)–%VRe(i4)–%VRe(i5) crossplots

Figure 12b offers a convenient way to estimate the %VRe of reservoir fluids using both the slope factor and the iC_4_/nC_4_ formulas (Eqs. 2 and 3). The maturity trend devised primarily based on the well-constrained Field G black-volatile oils and gas condensates represents the maturity evolution of indigenous (generative) unaltered fluids of thermal origin and exhibits an excellent match between VLH-based %VRe parameters: %VRe(SF), %VRe(i4) and %VRe(i5). The trend reasonably accommodates the entire unaltered fluid range examined from other fields, with a good general match observed between fluid types identified based on PVT data (exemplified in Fig. 1) and the maturity stages of petroleum formation identified based on source-rock kinetics^41^ (Fig. 12b,c).

The slope factor, iC_4_/nC_4_ and iC_5_/nC_5_ ratios all exhibit monotonically increasing evolutionary trends with thermal maturity for unaltered fluids. Deviations (or scatter) from the indigenous-fluid trend do occur for fluids altered by biodegradation, thermal cracking, or TSR cracking, as explained above. Respective deviation regions on the %VRe plots are highlighted in Fig. 12b,c. Corrections can, nevertheless, be made in most cases, where alteration affects one parameter but not the other. Biodegradation, for example, increases the %VRe(i4), with no apparent effect on the %VRe(SF) that can in this case be used to provide a more accurate estimate of thermal maturity. Thermal cracking, on the other hand, causes reversal to lower %VRe(SF) values, with no apparent reversal effect on the %VRe(i4) that can in this case be used to estimate thermal maturity. The shift of the thermally cracked fluids from the indigenous-fluid trend appears to be controlled by cracking intensity. A similar shift to subdued %VRe(SF), albeit in this case to a lesser degree, is observed for TSR-altered fluids, with a smaller effect on %VRe(i4). Lastly, phase separation of a single charge is expected to yield a single %VRe(i4) but variable %VRe(SF) values, as noticed for Field A (Fig. 12b), hence favouring the use of the former for assessing the thermal maturity of phase separation products of a single charge. The model of phase separation of a single gas charge in this field was also verified based on phase envelopes, fluid composition and other reservoir engineering considerations^50–53^. Less scatter in the altered fluids is observed on the %VRe(i5)–%VRe(i4) crossplot (Fig. 12c), allowing maturity estimations to be verified. Taking these and similar alteration-related caveats into account, the three %VRe crossplots collectively offer an excellent tool to estimate the correct thermal maturity of reservoir fluids and identify altered fluids, particularly when saturation pressures are considered, as discussed below.

##### %VRe and saturation pressure

**Figure 14:**
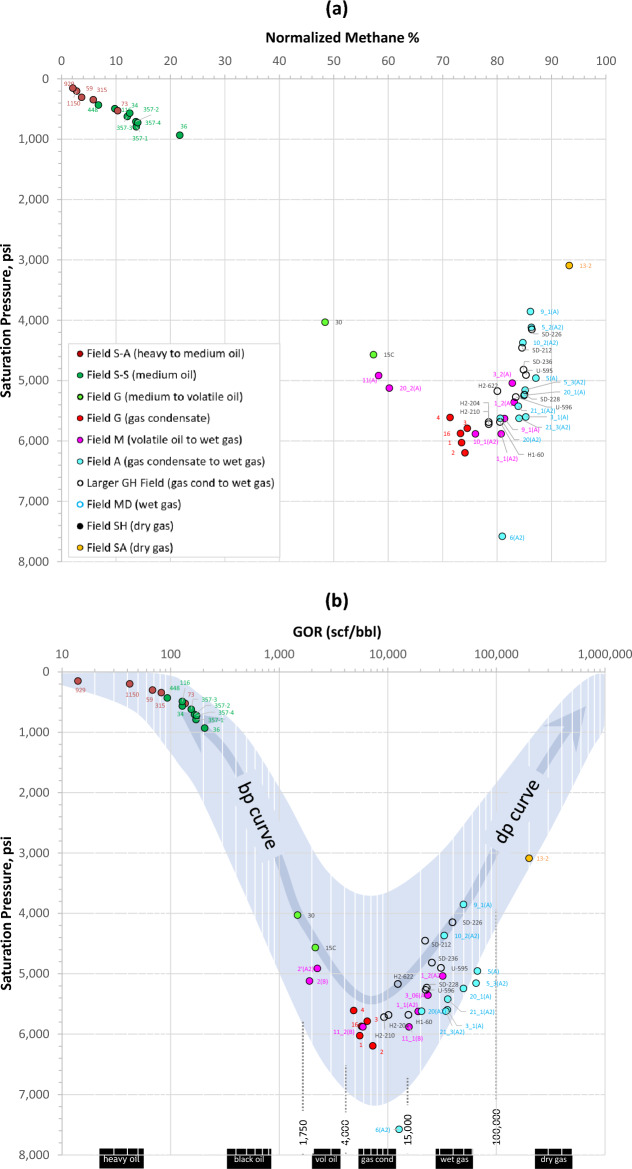
Relationships of (**a**) saturation pressure with normalized methane content, and (**b**) saturation pressure with GOR for all oil and gas fields discussed in this study. The blue band in panel b represents the global GOR–P_sat_ trend^18,19^. Methane normalized to hydrocarbon content only (C_1_/C_1-7+_).

Figure 14 illustrates the relationship between available saturation pressure (P_sat_), methane content and GOR across fields investigated. Consistent with the thermogenic origin and the predominant maturity control on fields S and G fluids, the P_sat_ increases steadily along the bubblepoint (bp) pressure trend with increasing methane, GOR and %VRe, from the heavy oil to gas condensate range. Leaner gas condensates and wet gases (fields GH, M and A) follow the dewpoint (dp) pressure trend, where the dewpoint pressure decreases while the methane and GOR increase. Note that the deeper, more-mature regions (U and SD) within the larger GH Field are characterised by higher methane and GOR values accompanied by lower P_sat_—testimony to their higher maturities compared to the shallower regions H1 and H2—despite greater alteration by TSR in the deeper regions. This highlights the importance of inspecting P_sat_ relationships when assessing thermal maturity.

Phase separation can lead to higher slope factors (and higher methane and GOR) that are not necessarily the result of more-mature charges. Comparable and overlapping maturity range for the wet gas in the northern part of Field M (wells 1, 3 and 11) and that in the 400-feet-shallower Field A indicates phase segregation of a single fluid system across both fields, with well 11 in Field M being the deepest and well 9 in Field A the shallowest. This and the almost identical C_2_/C_3_–C_3_/P_4_–P_4_/P_5_ compositional ratio profiles (Fig. 8) suggest that the wet gas across both accumulations may be in communication. Wet-gas connectivity was also proposed based on phase behaviour and structural restoration of the basin that agree with fluid redistribution across both structures following regional uplift and tilting to the east towards Field M^50–53^.

The dry gas (Field SA) along the dewpoint pressure curve in Fig. 14 represents a direct charge that was thermally cracked either in the source rock or remigrated from a deeper and hotter Paleozoic reservoir, as indicated by a VRe(i4) > 3% (Fig. 12b) and by the abovementioned VLH interrelationships, whereas the VRe(SF) is subdued (around 2%) due to thermal cracking (Fig. 12b).

## Data Availability

The datasets used and/or analysed during the current study are available from the corresponding author on reasonable request.
